# Visualization with Prediction Scheme for Early DDoS Detection in Ethereum †

**DOI:** 10.3390/s23249763

**Published:** 2023-12-11

**Authors:** Younghoon Park, Yejin Kim

**Affiliations:** Division of Computer Science, Sookmyung Women’s University, Seoul 04310, Republic of Korea; yejinkim@sookmyung.ac.kr

**Keywords:** blockchain, visualization, DDoS, polynomial regression, coefficient of determination

## Abstract

Blockchain technologies have gained widespread use in security-sensitive applications due to their robust data protection. However, as blockchains are increasingly integrated into critical data management systems, they have become attractive targets for attackers. Among the various attacks on blockchain systems, distributed denial of service (DDoS) attacks are one of the most significant and potentially devastating. These attacks render the systems incapable of processing transactions, causing the blockchain to come to a halt. To address the challenge of detecting DDoS attacks on blockchains, existing visualization schemes have been developed. However, these schemes often fail to provide early DDoS detection since they typically display only past and current system status. In this paper, we present a novel visualization scheme that not only portrays past and current values but also forecasts future expected system statuses. We achieve these future predictions by utilizing polynomial regression with blockchain data. Additionally, we offer an alternative DDoS detection method employing statistical analysis, specifically the coefficient of determination, to enhance accuracy. Through our experiments, we demonstrate that our proposed scheme excels at predicting future blockchain statuses and anticipating DDoS attacks with minimal error. Our work empowers system managers of blockchain-based applications to identify and mitigate DDoS attacks at an earlier stage.

## 1. Introduction

Recently, blockchain has become one of the essential data integrity solutions in many security-required applications [1,2]. These applications collect and manage data, which is safeguarded by blockchain-based security tools. In most cases, the data in such applications holds significant value, making financial or reputational damage a possibility in the event of a security breach. Therefore, while blockchain effectively preserves data integrity, additional security measures are necessary.

Within blockchain-based security systems, various types of attacks may emerge, including data leakage, theft of electronic wallets, and denial of service (DDoS) attacks. One common occurrence in these attacks is traffic flooding to the blockchain server, which can manifest not only during an attack but also before attacks commence. Attackers generate a large number of transactions to disrupt blockchain servers, resulting in massive traffic floods and gas consumption. Early detection is crucial in such scenarios, as identifying attack indicators in advance can minimize the extent of damage.

Several previous works have been developed to detect these types of attacks on blockchain-based systems. Most of these works involve measuring values related to attacks on blockchain systems [3]. For instance, Ji et al. measured the number of transactions to detect attacks on the Bitcoin network [4]. Similarly, Simon et al. proposed an intrusion detection system for the Ethereum network by analyzing mining rewards [5]. Additionally, various other previous works focus on detecting DDoS attacks on blockchain systems. However, these schemes merely present and anticipate values without visualizing the system’s status, making them less accessible to non-IT experts, such as system managers.

To address this issue, several visualization schemes that display the status of blockchain systems have been proposed. Many monitoring tools with visualization features for blockchains are available, such as Etherscan [6] and Ethviewer [7]. Furthermore, visualization tools for healthcare data management systems based on Hyperledger [7] and Logchain [8] have been introduced. Some applications that display the connection information of blockchain nodes have also been developed [9,10]. Nevertheless, these previous visualization schemes assist in attack detection but do not allow system managers to recognize attack indicators in advance due to their lack of predictive capabilities [11].

In this paper, we introduce a novel visualization tool for blockchain systems that includes a prediction scheme. Like existing visualization schemes, our proposed tool primarily provides visualization features that depict the current system status. In our research, we focus on the Ethereum private network as the blockchain and measure values relevant to DDoS attacks on the blockchain system, including gas consumption and the number of transactions in each block. We specifically anticipate the future number of transactions in each block and identify DDoS attacks by comparing the expected data with the actual data. Additionally, we measure the gas usage for block generation and compare it to the average to determine the occurrence of DDoS attacks. We validate the accuracy of our tool using various statistical methods.

The remainder of this paper is structured as follows: In Section 2, we first discuss the relevant studies, and Section 3 introduces the essential components of the proposed algorithms. Section 4 provides a system model, and Section 5 details our new visualization scheme. In Section 6, we demonstrate that our proposed scheme effectively detects DDoS attacks in the early stages. Finally, we conclude this paper in Section 7.

## 2. Related Works

In this section, we review previous works related to visualization schemes for blockchain.

### 2.1. Visualization for Blockchain-Based Systems

While there are several security visualization schemes for general network systems, those specifically designed for blockchains have been relatively scarce. In the early stages, graph-based visualization schemes for cryptocurrency were proposed. Works such as [12,13,14] focused on data analysis and forensic investigation of transactions but did not consider security-related values. Tharani et al. [9] later proposed an advanced version that detects malicious transactions in Bitcoin, including ransomware and illicit advertisements. Similarly, refs. [15,16] presented visualization schemes for Ethereum, addressing fraud and vulnerability issues in transactions and smart contracts.

Another type of visualization scheme involves displaying network topologies. Delgado-Segura et al. introduced a technique for finding the Bitcoin network topology using orphan transactions [17], and Johnson et al. proposed a scheme that visualizes the mining pool and detects DDoS using a game-theoretic approach [18]. Miller et al. developed an advanced topology for the mining pool, highlighting influential nodes [19]. Additionally, Maeng et al. visualized the topology for the Ethereum network [20]. However, these visualization schemes primarily depict the current status without predicting future values.

### 2.2. Prediction for Blockchain Values

Various monitoring tools exist for blockchain systems, such as [21,22]. In [23], the authors presented a monitoring system for Bitcoin that detects illegal proof-of-work blockchains. Chen et al. proposed a monitoring technology that detects Ponzi schemes on public blockchains. Additionally, monitoring tools for smart contracts in Ethereum were provided [24,25]. However, these tools lack a prediction scheme for fast attack detection.

Research on predicting the future status of blockchains has also been conducted. Zheng et al. defined metrics for measuring the current status of the blockchain [3], and Ji et al. proposed predicting the number of transactions in the blockchain based on machine learning [4]. Simon et al. anticipated mining rewards using polynomial regression for Ethereum. However, they did not consider time in predicting future values, possibly because the studied blockchains were public [5].

## 3. Backgrounds

### 3.1. Blockchain Structure

Blockchain is a chain of blocks that allows for the secure storage of all committed transactions using shared and distributed networks [26,27]. To achieve this, decentralization techniques and robust cryptographic schemes are employed. These measures guarantee the integrity of the data within the blockchain, ensuring that data cannot be tampered with illegally. Blockchain is characterized by four key concepts: Decentralization, Persistency, Anonymity, and Auditability [27,28].

Decentralization is a critical feature that safeguards data within the blockchain system. In traditional security schemes, important data are often managed in a centralized manner, where data are stored in a central database, and keys for encryption/decryption and digital signatures are generated by a central server. Additionally, security policies and key management methods are determined by the server, and users obtain keys from the server and must adhere to its rules.

However, centralized systems have inherent problems, including a single point of failure and concentration of power:

- Single point of failure: In centralized security systems, essential information, including security-required data and keys, is stored on a central server. If an attacker breaches the central server and gains control, they can illegally access and modify the stored data. Furthermore, an attack on the central server can lead to a complete system shutdown. Consequently, the central server becomes a prime target for attackers, and real-world security breaches on central servers are well-documented.
- Concentration of power: In most security systems, the central server has administrator authority. Therefore, the system manager possesses unrestricted access to data, enabling them to view and modify data as they see fit. Even without malicious intent, if the central server is compromised, all data on the server is at risk. These issues arise because administrative power is concentrated solely within the central server.

To address these limitations of traditional security systems, blockchain technology has been proposed. Blockchain ensures the perfect integrity of stored data through decentralization and robust cryptographic measures. Due to these properties, blockchain is now widely employed in systems where data integrity is paramount.

In a blockchain-based security system, data are stored in a decentralized manner across multiple nodes (assuming there are *n* nodes) instead of relying on a single server. This means that even if a user managing one node attempts to illegitimately alter data, the integrity of the data remains intact because the data on the other n−1 nodes remain unaltered. For an attacker to modify blockchain data, more than half of the *n* nodes must be compromised. As the number of nodes grows and they are distributed globally, it becomes increasingly challenging for attackers to compromise more than half of the nodes.

However, the blockchain system must also ensure data integrity even when the number of nodes is small. To achieve this, an additional security measure is employed: the consensus algorithm. In blockchain systems, data or their hash values are stored within each block. To prevent illegal modifications, each block must be linked to the blockchain using a predetermined algorithm known as the consensus algorithm. Various consensus algorithms exist, such as Proof of Work (PoW) and Proof of Stake (PoS). Connecting blocks with the consensus algorithm is often a complex process. Thus, if an attacker attempts to modify data within the blockchain, they must regenerate blocks from the point of data modification to the end block using the challenging consensus algorithm. This task is nearly impossible, ensuring the perfect integrity of the data.

### 3.2. DDoS on Blockchain

The three fundamental elements of security are confidentiality, integrity, and availability. As previously mentioned, blockchain effectively ensures the integrity of stored data. Furthermore, in most blockchain-based systems, additional encryption and key management schemes are implemented to enhance confidentiality. Consequently, most attacks on blockchain systems aim to disrupt availability, typically through Distributed Denial of Service (DDoS) attacks.

In the context of blockchain, the most common method for launching a DDoS attack involves flooding the network with transactions. Legitimate users generate normal transactions to input data into each block or send cryptocurrency to other users. However, in a DDoS attack, spam or fake transactions are sent to the blockchain server, leading to the rejection of normal transactions or even a system shutdown.

Several examples of DDoS attacks on blockchain systems have been documented. A DDoS attack on the Solana blockchain occurred for 17 h in December 2021 [29], and another attack took place in January 2022 [30]. Multiple attacks on the Ethereum blockchain have also been reported [31,32]. Table 1 presents well-known examples of DDoS attacks on blockchain systems.

Numerous solutions exist to prevent or mitigate DDoS attacks on blockchain systems. These solutions can be categorized into proactive and reactive methods. Proactive methods include retaining sufficient storage, memory, and network bandwidth, among other strategies. Reactive methods aim to mitigate DDoS attacks after they occur. Within the realm of reactive solutions, visualization tools that display the blockchain server’s status have been developed to detect attacks earlier. However, existing visualization tools only reveal the current state of the blockchain, which may not be sufficient for rapid DDoS response. In this paper, in addition to the current status, we present future values for the blockchain status to enable quicker responses to DDoS attacks.

## 4. System Architecture

**Figure 1:**
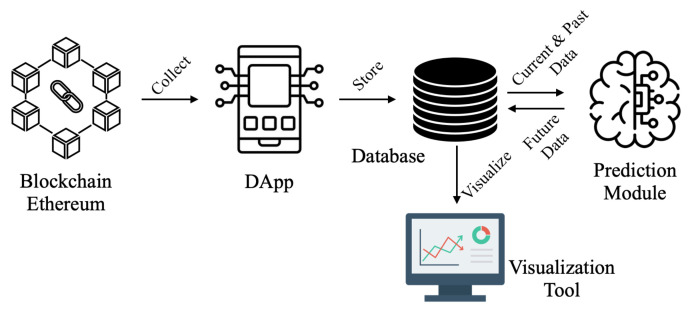
Overview of the Entire System.

In this section, we introduce the entire system architecture. Figure 1 provides an overview of the entire system. The blockchain server generates various data while it runs, and the Decentralized Application (DApp) collects the data essential for predicting attacks. The collected data are stored in a database, and the prediction module calculates the future values for possible attacks. The results of the predictions are also stored in the database. Finally, the collected and predicted data are presented in plots to anticipate possible attacks using the visualization scheme.

Now, let us describe the detailed roles of the components.

- Blockchain Server: The blockchain server is a powerful computer that runs a blockchain, generating various data. This server must be connected to the internet. Among the data generated, some are related to the blockchain, and others are not. The DApp, which will be explained in the next paragraph, collects data that are essential for predicting future attacks. In this paper, we use Ethereum for a private blockchain and employ Geth to build the blockchain network. The collected data are closely related to attack attempts. For example, gasUsed represents the amount of gas used to create a new block.
- Decentralized Application (DApp): The DApp is a web application that operates directly on the blockchain or communicates directly with the blockchain RPC interface as a decentralized client [33]. The DApp’s purpose in this paper is to collect blockchain information related to various attacks. The collected data for this paper are listed in Table 2. Additionally, for this work, we build the DApp with Node.js v18.16.1 and Web3 v0.20.6.
- Database: After the DApp collects data for intrusion detection, it sends the data to the database. The database collects information from the initial state to the current state of the blockchain. Furthermore, this database stores future data expected by the prediction module. Afterward, the collected and expected data are delivered to the monitoring tool to visualize the current state and check for any attacks.
- Prediction Module: The prediction module in this work aims to forecast the future values based on the current and past values on the blockchain server. The prediction module receives the current and past values for the blockchain server’s status from the database. After the prediction, the future values are sent back to the database. The detailed procedure for the prediction module will be shown in Section 5.
- Monitoring Tool: After the database collects not only past and current data but also expected future data, it sends them to the monitoring tool. The monitoring tool displays this data through plots over time. Additionally, our monitoring tool issues a warning when it suspects an intrusion attack based on abnormally high future values. In this work, we build the monitoring tool with Node.js v18.16.1 and Grafana v9.2.1.

## 5. Proposed Detection Scheme

In this subsection, we will explain the detailed procedures of the system. The process can be classified into three steps. First, data collection from the Ethereum blockchain and saving the extracted data to the Maria DB are operated. Then, to use multiple linear regression to predict the number of transactions and the amount of gas, we selected particular data based on the degree of correlation. Finally, we visualize the current data in a solid line and the future data in a dotted line.

### 5.1. Data Collection

Several models have been proposed for monitoring the blockchain system. Sayadi et al. detect abnormal electronic transactions in the Bitcoin system [34], and they extract Bitcoin transaction data, block size, difficulty, hash rate, transaction volume, median time for transaction confirmation, and the number of unique Bitcoin transactions per day. In addition, in [35], the authors extract the number of transactions and the volume of gas consumption.

We determine the data to be extracted from the blockchain by referring to these previous studies and select data for future data prediction additionally. For the data collection process, we use the Web3 Node.js library to connect to the Ethereum node via HTTP. We extract blocks and transaction data from Ethereum using the Web3.eth.getBlock() function. Finally, we collect the data listed in Table 2: blockNumber, difficulty, gasUsed, size, timestamp, totalDifficulty, and transactionNumber. Among them, gasUsed and transactionNumber are used to predict attacks earlier.

The collected data are delivered to a database. In our work, we employ MariaDB to store them. The saved data are used for forecasting future values and will be displayed in plots by a monitoring tool.

### 5.2. Prediction with Future Values

DDoS attack on blockchain is carried out as an attack through transaction flooding. By producing a lot of spam or false transactions, the system needs more time to validate false transactions, which results in a network overload. Therefore, for anomaly detection of DDoS attacks, we focus on the number of transactions per block, transactionNumber. In this paper, we detect the DDoS attack by predicting the future transactionNumber values.

In addition, gas consumption in the Ethereum refers to a blockchain’s transaction fee. In this work, we collected it as a name of gasUsed. This gas is paid to network validators for the requested services to the blockchain. If the network is congested, gas prices might be high and thus gas consumption will increase. Therefore, we focus on not only the number of transactions but also the gas consumption to check the traffic. In this work, we predict the DDoS attack by measuring gasUsed value.

We first choose the values that are used to predict the future values of transaction-Number. In terms of the future transactionNumber, we select current and past difficulty, gasUsed, size, transactionNumber, and time value. Of course, the current and past transactionNumber values influence the future transactionNumber value. In addition, transactionNumber also depends upon the time, because in most cases, more transactions emerge in the daytime, and the amount of the transactions decreases at night. Then, we demonstrate that the other properties difficulty, gasUsed, and size is also strongly related to transactionNumber by experiments.

**Figure 2:**
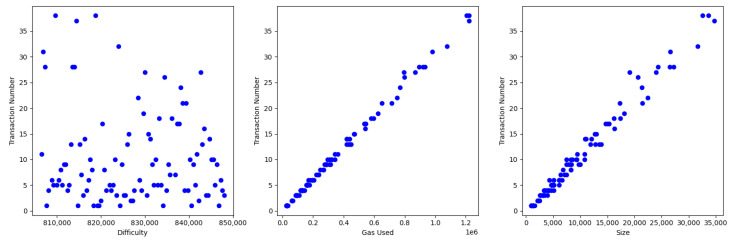
Scatter plots for transactionNumber subject to difficulty, gasUsed, and size.

Figure 2 indicates scatter plots for transactionNumber subject to difficulty, gasUsed, and size. In addition, as Table 3, we calculate the correlations between transactionNumber and difficulty, gasUsed, and size. From them, we conclude that gasUsed and size are strongly related to transactionNumber. In addition, although difficulty has a relatively lower correlation to transactionNumber than other factors, the correlation value is near 0.5, so we can conclude that difficulty has also a relation to transactionNumber.

Using the above four blockchain values and time, we predict the future transaction-Number. In the polynomial regression, for modeling, the prediction module gets the four blockchain value from MariaDB and calculates the coefficients of the polynomial regression in Figure 1. After the modeling is down, the prediction module collects the current and past transactionNumber, difficulty, gasUsed, and size, and measures current time. With these information, the module calculates the future transactionNumber using the polynomial regression.

**Figure 3:**
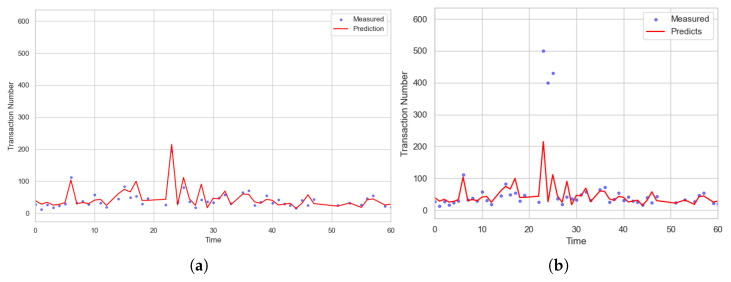
Visualization for transactionNumber for 1 h; (**a**) Case for normal transactions; (**b**) Case for DDoS attack.

Figure 3 indicates the visualization results for an hour that show not only the past and current transactionNumber, but also the future values. The blue dots mean the current values and the red lines are the graphs for future values. Figure 3a is the result for the normal case. As the figure, the predicted values are nearly matched to the measured ones. From this, our proposed scheme predicts transactionNumber well. In addition, for the second case, we observe a DDoS attack on the blockchain system at 20 min. As Figure 3b there are gaps between the blue dots and red line around 20 min. By using this, we can predict the DDoS early.

**Figure 4:**
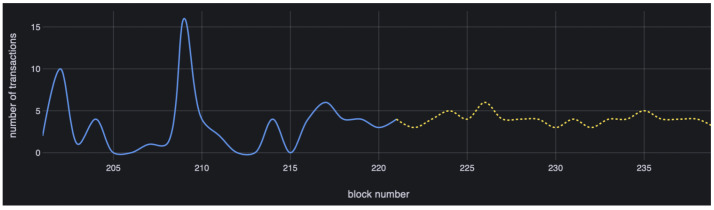
Graph for past, current and future values of transactionNumber.

Figure 4 is the graph for transactionNumber. The solid line is the graph for current and past values, and the dotted line is one for future values. We draw this graph using Grafana with the data in MariaDB. By using this graph, we can expect the near future transactionNumber value, so we will be able to detect early when a DDoS attack begins.

### 5.3. Detection with Statistical Method

In the previous subsection, we provide a prediction scheme by expecting transaction-Number with a visualization tool. However, gaps between the expected value and the actual measured value can occur due to various normal reasons. Therefore, a false positive can be determined. To overcome this misjudgment, we supplement one more detection scheme using a statistical method.

In this paper, we provide another detection scheme using the interquartile range (IQR) method with gasUsed value. In Ethereum, ’gas’ refers to the computational cost of processing transactions on the network. It is consumed when a transaction is submitted or when cryptocurrency is transmitted to other users. Therefore, if the amount of the gas becomes much larger than the average amount, we can expect that a DDoS attack will occur. In fact, if the gas is consumed more than the expected amount, the transaction or sending the cryptocurrency can become invalid. Therefore, measuring the usage of gas is another important concern in predicting a DDoS attack on the blockchain.

In this work, we first measure the usage of gas for several days and calculate the average amount. Next, we use the Interquartile Range (IQR) method to detect DDoS attacks. In our experiment, the top quarter amount of the gas usage is 4,566,790, so we determine that there is a DDoS attack on our blockchain system when the amount of gas usage is larger than that value. We demonstrate the validity of this method for detecting DDoS attacks with fewer false positives and false negatives in the next section.

## 6. Discussion

In this section, we demonstrate that our proposed prediction scheme, utilizing both visualization and statistical-based methods, accurately detects DDoS attacks. To assess the performance of the prediction scheme using the visualization tool, we examine the discrepancies between the expected and real values. Additionally, we verify the validity of the statistical method by calculating accuracy and F1 score.

We will first discuss the accuracy of the prediction scheme. As previously mentioned, to evaluate the effectiveness of our prediction scheme, we calculate the difference between the measured values and the expected values. This involves computing the Mean Squared Error (MSE) and the coefficient of determination ($R^{2}$). As our proposed scheme predicts more accurate values, MSE must decrease. In addition, because the MSE is in the numerator of the equation of $R^{2}$, $R^{2}$ approaches to 1 as our work expects well. Anticipating accurate future values implies that our scheme effectively detects normal cases, leading us to conclude that the likelihood of false positives in DDoS detection is low.

The MSE is calculated as follows:

$$MSE=\frac{1}{2}\sum_{i=0}^{n}{\left(y_{i}-{\hat{y}}_{i}\right)}^{2},$$

where *n* is the number of measurements, $y_{i}$ is the measured *i*-th value, and ${\hat{y}}_{i}$ is the expected *i*-th value. In the prediction scheme, we calculate the expected value of transactionNumber, making $y_{i}$ represent the *i*-th transactionNumber, and ${\hat{y}}_{i}$ represent the expected value. To obtain the MSE, we run the Ethereum server for one hour, measure the real transaction-Number, and calculate the expected transactionNumber. This experiment is conducted to demonstrate that our proposed prediction scheme performs well under normal circumstances, with normal transactions.

Additionally, we calculate the coefficient of determination ($R^{2}$). We collect data in the same manner and compute the following equation:

$$R^{2}=1-\frac{\sum_{i=1}^{n}{\left(y_{i}-{\hat{y}}_{i}\right)}^{2}}{{\left(y_{i}-\bar{y}\right)}^{2}},$$

where $\bar{y}$ is the average of $y_{i}$ values. In other words, $\bar{y}=\frac{1}{n}\sum_{i=1}^{n}y_{i}$.

Table 4 displays the MSE and the Coefficient of Determination for transactionNumber. Smaller MSE values indicate more accurate predictions. The calculated MSE is 0.0215, significantly smaller than the average value of transactionNumber. As shown in Table 3, the average number of transactions in each block is approximately 30. This low MSE demonstrates the accuracy of our polynomial-regression-based prediction scheme. We can further support this by calculating the coefficient of determination. As the scheme makes more accurate predictions, the coefficient of determination approaches 1. In Table 4, the $R^{2}$ value is 0.9999, indicating that the prediction scheme works effectively.

Now, we propose an alternative scheme that predicts DDoS attacks by monitoring gasUsed using a statistical method. To validate the performance of this second scheme, we calculate True Positives (TP), False Positives (FP), True Negatives (TN), and False Negatives (FN) to derive metrics like Accuracy, Precision, Recall, and F1−Score. These metrics are calculated as follows:

$$Accuracy=\frac{TP+TN}{TP+FP+TN+FN},\;Precision=\frac{TP}{TP+FP},$$

$$Recall=\frac{TP}{TP+FN},\;F1-Score=2\times \frac{Precision\times Recall}{Precision+Recall}.$$

These metrics, all ranging from 0 to 1, approach 1 as the prediction scheme more accurately identifies DDoS attacks.

From the results shown in Table 5, our statistical-based DDoS prediction scheme successfully detects attacks with a high degree of accuracy.

## 7. Conclusions

In this paper, we have presented a DDoS prediction tool that leverages both visualization techniques and a statistical method. Our prediction tool comprises a visualization module with value expectation techniques and a detection scheme based on a statistical method. The visualization module provides a clear presentation of the current and past values of the number of transactions in each block, as well as the expected future values. To accomplish this, we employ polynomial regression to forecast these future values. In addition, our statistical method focuses on measuring the amount of gas used in creating each block. By calculating the gap between the current amount and the average amount, we can detect DDoS attacks at an earlier stage.

Our work demonstrates that the prediction scheme in the visualization tool accurately predicts future values, as indicated by the small Mean Squared Error (MSE). Furthermore, we have shown that the statistical method can effectively detect DDoS attacks with a low rate of false positives and false negatives. With the implementation of our tool, we can identify DDoS attacks on blockchain systems at an early stage, thereby mitigating potential damage caused by such attacks.

## Figures and Tables

**Table 1:** Well-known Examples of DDoS on Blockchain.

Date	Blockchain	Attack
February 2021	EXMO	Service stopped for 5 h
December 2021	Solina	Network was offline for 17 h
December 2021	Arbitrum	Network was offline for 45 min
2022	Solina	DDoS was occurred for 3 times

**Table 2:** List of Collected Data from Ethereum Server.

Collected Data	Meaning
blockNumber	Each block’s number
difficulty	An integer value how difficult it is to mine a block
gasUsed	A total gas used by all transactions in a block
size	The size of a block
timestamp	An unix timestamp when a block was collected
totalDifficulty	An integer value of how difficult it is to mine whole blocks from initial block to the current block
transactionNumber	The number of transactions in a block

**Table 3:** Correlations between transactionNumber and difficulty, gasUsed, size.

	difficulty	gasUsed	size
transactionNumber	0.4835	0.9996	0.9998

**Table 4:** Mean Squared Error and Coefficient of Determination for transactionNumber.

	MSE	$R^{2}$
transactionNumber	0.0215	0.9999

**Table 5:** Performance Evaluation of Statistical Method.

	Accuracy	Precision	Recall	F1-Score
Value	0.8667	0.8	0.8	0.8

## Data Availability

Data are contained within the article.
